# A novel donor site mutation in LMNA gene leading to severe form of Dilated Cardiomyopathy in a proband of a family from Bihar, India

**DOI:** 10.1186/1755-8166-7-S1-P35

**Published:** 2014-01-21

**Authors:** Soumi Das, Amitabh Biswas, Mitali Kapoor, Sandeep Seth, Balram Bhargava, Vadlamudi Rao

**Affiliations:** 1 Department of Anthropology, University of Delhi, Delhi, India; 2 Department of Cardiology, AIIMS, India

## Background

Dilated Cardiomyopathy (DCM) is poorly understood in terms of their mechanistic pathways. It may lead to sudden cardiac death with a prevalence rate of 0.04-0.2%. The cause of DCM is still unknown and referred to as Idiopathic Dilated Cardiomyopathy. There are many candidate genes associated with the DCM but most of the mutations are found in the LMNA and MYH7 genes.

## Methods

The proband underwent Echocardiography and ECG to confirm the diagnosis. 5ml blood was collected and DNA was extracted using Phenol-chloroform method. The hot spot regions exon 23 of MYH7 gene, exon3 and exon 4 along with the intron3 of LMNA gene were sequenced using Sanger sequencing (ABI 3730xl). ACE 287bpI/D and *TNNT2*5bpI/D polymorphisms were also genotyped. *In silico* analysis of this novel mutation by using softwares, Human splicing finder (HSF) and MaxENT to understand the effect of mutation on splicing. The study was ethically approved by Institutional committee and informed written consent was taken from all participants.

## Results

The proband aged 50yrs diagnosed with DCM, age of onset 45yrs, showing severe symptoms such as dyspnea, palpitation, fatigue and pedal edema under NYHA III classification showing dilated LV with EF 30%. Proband’s mother died due to heart problem but was not clinically confirmed for DCM and his sister had a suspicious death. The son of the proband aged 24 years has the same LMNA mutation but is asymptomatic presently.

A Novel donor splice site mutation G>C transversion in intron3 of LMNA gene and a synonymous mutation (C>T at codon 923) was found in MYH7 gene in proband. ACE and *TNNT2* polymorphisms showed a heterozygous (ID) and homozygous (II) genotypes respectively. *In silico* analysis by HSF and MaxENT shows that the elimination of natural donor splice site leading to the use of a cryptic donor site which is located 7 nucleotide upstream of native splice site.

## Conclusion

As reported in previous studies, LMNA gene mutations are associated to the severe form of DCM. From the above results, it may be concluded that the severe form of the disease is due to the splice site mutation.

